# Supplementary material for: Complexome profiling of the Chlamydomonas psb28 mutant reveals TEF5 as an early PSII assembly factor
Source: Plant Cell. 2025 Mar 17;37(6):koaf055. doi: 10.1093/plcell/koaf055 (PMC12164590; doi:10.1093/plcell/koaf055)
Supplement: koaf055_Supplementary_Data [file koaf055_supplementary_data.zip › psb28_interactive.zip!/plots/intensity/qualityPlots/peptides/SOUL3_Cre16.g666550.t1.1.html]

### Cre16.g666550.t1.1

Trivial name: SOUL3  
SeqCoverage [median]: 19.20  
Mass: 47.67 kDa  
  
 

| Sequence | MissedCleavage | Mass [Da] | wtIntensity | muIntensity | Score |
| --- | --- | --- | --- | --- | --- |
| AYEPYTVAETSMGSGAGPASGAGFSDLAR | 0 | 2819.27 | 0.00 | 1236.21 | 58.26 |
| DGYKPAVGYQLAR | 1 | 1436.74 | 1391.10 | 5758.61 | 98.11 |
| YADLAALPAPSDPR | 0 | 1455.74 | 318.07 | 6623.74 | 114.86 |
| YNDPSTPPGIR | 0 | 1215.59 | 0.00 | 1414.89 | 78.94 |
| YNDPSTPPGIRR | 1 | 1371.69 | 252.32 | 786.20 | 87.57 |
| YSPDITFEDPITK | 0 | 1524.73 | 902.14 | 0.00 | 80.98 |
|  |  |  | 2863.6 | 15819.6 |  |
